# Phytochrome-mediated light perception in dodders drives haustorium development through epigenetic mechanisms

**DOI:** 10.1093/pcp/pcag043

**Published:** 2026-04-02

**Authors:** Thomas Bawin, Andreas Evenstad, Alena Didriksen, German Martinez, Kirsten Krause

**Affiliations:** Department of Arctic and Marine Biology, UiT The Arctic University of Norway, Framstredet 39, 9019 Tromsø, Norway; Department of Arctic and Marine Biology, UiT The Arctic University of Norway, Framstredet 39, 9019 Tromsø, Norway; Department of Arctic and Marine Biology, UiT The Arctic University of Norway, Framstredet 39, 9019 Tromsø, Norway; Department of Plant Biology, Uppsala BioCenter, Swedish University of Agricultural Sciences and Linnean Center for Plant Biology, Almas Allé 8, 750 07 Uppsala, Sweden; Department of Arctic and Marine Biology, UiT The Arctic University of Norway, Framstredet 39, 9019 Tromsø, Norway

**Keywords:** parasitic plants, light signalling, haustorium development, epigenetic regulation, phytochrome pathways

## Abstract

*Cuscuta* species, parasitic plants with minimal photosynthetic capacity, rely on light cues to locate hosts and initiate infection. Unlike nonparasitic plants, they exhibit a reversed shade response, growing towards low red:far-red (R:FR) light typical of dense vegetation. We investigated how R and FR light modulate haustorium development, gene expression, and epigenetic reprogramming in *Cuscuta campestris*. FR-enriched conditions promoted coiling and haustorium initiation, while red light suppressed parasitic behaviour. Phytochromes B1 and B2 displayed opposing transcriptional responses to light quality, suggesting a modified light perception mechanism. Transcriptome analyses revealed further that FR light triggered the differential expression of over 5000 genes, including those linked to auxin and cytokinin signalling, cell wall remodelling, and organogenesis. Gene co-expression networks identified phytochrome B2 and a Fhy1/Fhl regulator of phytochrome A as possible central hubs associated with chromatin remodellers, histone modifiers, and RNA-directed DNA methylation components. Small RNA profiling indicated stable global sRNA populations across treatments, with shifts in the expression of specific miRNA families, affecting a subset of light-responsive genes. Our findings demonstrate that FR light perception in *C. campestris* engages both transcriptional and epigenetic regulation to drive haustorium development, reflecting adaptations in light signalling cascades that underpin its parasitic lifestyle.

## Introduction

The ability of plants to perceive and respond to light is crucial to optimize their performance and ensure their survival. Light quantity and quality, indeed, indicate the presence and position of neighbours, and plants strive to escape shading and competition for light via phototropic growth (Ballaré and Pierik 2017). For this process, some areas of the light spectrum, such as red and blue light, are more important than others. For parasites from the genus *Cuscuta* (dodders), the ability to interpret light signals is at least equally critical even though their thread-like shoots exhibit at best a residual photosynthetic activity (Schneider et al. 2025). The reason is that these parasites, being devoid of proper leaves and roots, are entirely dependent on a host from the initial seedling stage onwards (Shimizu and Aoki 2019, Bawin and Krause 2024, Balios et al. 2025). Both seedlings and older shoots wind in tight coils around the aerial parts of potential hosts upon their contact and develop infective structures (called ‘haustoria’) that penetrate the host tissues and connect to their vasculature. This process enables dodders to sequester water, inorganic salts, and organic compounds, creating a nutrient sink (Westwood et al. 2010, Kim and Westwood 2015). The fact that light is an essential component of parasitism in *Cuscuta* species was discovered before any molecular details of the infection process were unravelled. Seedlings were demonstrated to direct their growth towards far-red (FR) light and use the low red:FR (R:FR) signature of the green foliage of nearby hosts to forage for the potentially most rewarding ones (Orr et al. 1996, Benvenuti et al. 2005, Johnson et al. 2016, Smith et al. 2021). FR and blue lights were further shown to be effective in potentiating coiling as well as haustorium development in seedlings with an additional tactile signal, and that effect could be reversed by red light (Lane and Kasperbauer 1965, Tada et al. 1996, Haidar et al. 1997, Furuhashi et al. 1995, 2021, Kaga et al. 2020, Bawin et al. 2022, Yokoyama et al. 2023, 2025). Once parasitism is established, a rapid elongation of the attached shoot can be observed, followed by the development of side shoots that spread onto other parts of the hosts and to nearby hosts, again guided by the quality of the light (Olsen et al. 2016, Yokoyama et al. 2023).

Plants sense different wavelengths of light with a complex array of receptors. In *Arabidopsis thaliana*, five phytochromes (AtPhyA–E) are mostly responsive to R and FR waves, while cryptochromes (Cry1–2) and phototropins (phot1–2) are the main characterized sensors of blue light (Jing and Lin 2020). These receptors mediate light perception and activation of downstream signalling events that constantly regulate the plant transcriptome with additive, synergistic, or antagonistic effects. Transcription factors such as the master regulator ELONGATED HYPOCOTYL 5 (HY5) and several PHYTOCHROME INTERACTING FACTORS (PIFs) integrate the signals and translate them to differential growth responses between tissues and organs (Jing and Lin 2020, Cheng et al. 2021, Huber et al. 2021). The perception in *Cuscuta* of R and FR lights on one hand and blue light on the other hand was attributed to phytochromes and cryptochromes, respectively, as a change in the R:FR ratio was correlated with the ratio of active phytochromes, and as phytochrome inhibition did not influence the effect of blue light (Lane and Kasperbauer 1965, Furuhashi et al. 1997, Haidar and Orr 1999, Haidar 2003). In *Arabidopsis*, the movement away from shade is mediated by an asymmetrical inactivation of AtPhyB across the stem and a local accumulation of PIFs on the same side (Lorrain et al. 2008, Ballaré and Pierik 2017), leading ultimately to a unilateral growth promotion. Interestingly, in *Cuscuta* FR-rich shade conditions induce the opposite reaction, with slower growth observed on the proximal stem side, leading to a bending and ultimately coiling reaction towards and around the shade-providing host. Signal transduction via phytohormones such as auxin and cytokinin, together with ion fluxes, was suggested to promote parasitism downstream of light perception (Ramasubramanian et al. 1988, Haidar et al. 1998, Furuhashi et al. 2011, 2021, Haidar and Shabala 2015, Pan et al. 2022). Due to a fairly recent whole-genome duplication (Vogel et al. 2018, Segawa et al. 2026), *Cuscuta campestris* contains two copies of five phytochromes in its genome*.* In contrast, some phytochrome-associated genes like *HMR* and *SIG2*, together with many members of their coregulated gene clusters, were lost in *C. campestris* (Vogel et al. 2018), suggesting that the road to *Cuscuta* parasitism altered the signal relay process and not the perception of R or FR light. Recently, Yokoyama et al. (2025) showed that specifically one form of the phytochrome genes in *C. campestris* forms a distant subclade of the phytochrome B group (CcPhyB2) and is regulated in a light-dependent and haustoriogenesis-correlated manner, while other phytochrome genes (CcPhyA, B1, C, and E) do not show light-dependent expression. How light signals, however, are translated at the molecular level into an infective behaviour, and how the set of molecules playing a role in shade avoidance has been modified to allow for a different response, remains a long-standing, yet largely unanswered intriguing matter (Furuhashi et al. 1997, 2011, Jhu and Sinha 2022, Bawin and Krause 2024, Balios et al. 2025).

Epigenetic remodelling adds another level of plasticity downstream of light perception. Changes in chromatin architecture, histone modifications, and expression of noncoding RNAs are among the best-known mechanisms of epigenetic reprogramming that are targeted by light receptors, with possible light-specific effects (Torres et al. 2019, Jing and Lin 2020, Patitaki et al. 2022). In *A. thaliana*, AtPhyA activation in the dark, or repression by light, correlates with changes in transcript levels and alteration of histone marks at the PHYA locus, enabling rapid and reversible responses (Jang et al. 2011). AtPhyB was further suggested as a central component that influences nucleus size and chromatin condensation levels in response to light conditions, possibly via HISTONE DEACETYLASE 6 and METHYLTRANSFERASE 1 (MET1) (Tessadori et al. 2009, van Zanten et al. 2010, To et al. 2011, Snoek et al. 2017). Histone marks and DNA methylation are indeed highly interdependent, with important roles in regulating genome stability, chromatin compartmentalization, and gene imprinting and expression (Zhang et al. 2018). DNA methylation can be established *de novo* by the RNA-directed DNA methylation pathway (RdDM), which uses small RNA (sRNA) guides produced by DICER-LIKE 3 from the processing of RNA-DEPENDENT RNA POLYMERASE 2–amplified RNA POLYMERASE IV transcripts. Through molecular cascades, it leads to cytosine methylation in three possible contexts (CG, CHG, and CHH, with H being any base except G) at complementary target loci by DOMAINS REARRANGED METHYLTRANSFERASE 2. DNA methylation is maintained by MET1 and CHROMOMETHYLASE 2 and 3 (Zhang et al. 2018, Erdmann and Picard 2020). Many of the studies exploring the link between light perception and DNA methylation, however, focused on UV light, which through the UVR8 receptor triggers a loss of DNA methylation via inhibition of DRM2-mediated DNA methylation and a transcriptional de-repression of transposable elements (TEs) (Jiang et al. 2021, Patitaki et al. 2022). More recently, it was shown that phytochrome-mediated regulation of gene expression in tomato during fruit development and ripening correlates with changes in DNA methylome and sRNAome profiles, suggesting the involvement of the RdDM pathway in a light-dependent manner (Bianchetti et al. 2022).

Given a shade response that is diametrically opposite in *Cuscuta*, it is likely that downstream targets of the phytochrome signalling cascade or its regulation have changed on the evolutionary way to its parasitic lifestyle, and that this could have included DNA modification patterns. Here, we performed an integrative analysis of the dynamics of DNA methylation, sRNA accumulation, and gene expression in response to (far-)red light during the early steps of haustorium development in *C. campestris*. We show that gene networks downstream of phytochromes involve both (sRNA-mediated) epigenetic and transcriptional factors leading to altered methylation and expression profiles of haustorium-associated genes.

## Results

To study the effect of R and FR light regimes on *Cuscuta* behaviour in more detail, we first executed some basic host infection experiments with detached apical shoot segments of *Cuscuta* exposed to different light regimes*.* In brief, a marked decrease of developed haustoria on attached shoots was seen when the proportion of RL was increased (Supplementary Data S1), corroborating earlier reports (Johnson et al. 2016) with indications of a dose dependency on the relative amount of RL. Reversing the R:FR ratio and exposing the shoots to FR-supplemented light, in contrast, clearly rescued haustorium production. While this general phenology and behaviour were robust and always visible, the variation in terms of the number of haustoria or parasite net growth was substantial between experiments done in different weeks and with different host batches. During all the following experiments targeted to unravel light-induced changes at DNA and RNA level, the live hosts were therefore exchanged for wood sticks to minimize the natural biological variation between individuals to just one interaction partner. For illumination conditions, the opposing light regimes of 16 h white light, supplemented with either R or FR light and an additional 2 h of R or FR light only before a 6-h-long night were chosen [see Supplementary Data S1 (light regimes B and C), Supplementary Fig. S1].

### Coiling under high far-red to red light ratios is accompanied by considerable transcriptional reprogramming

**Figure 1:**
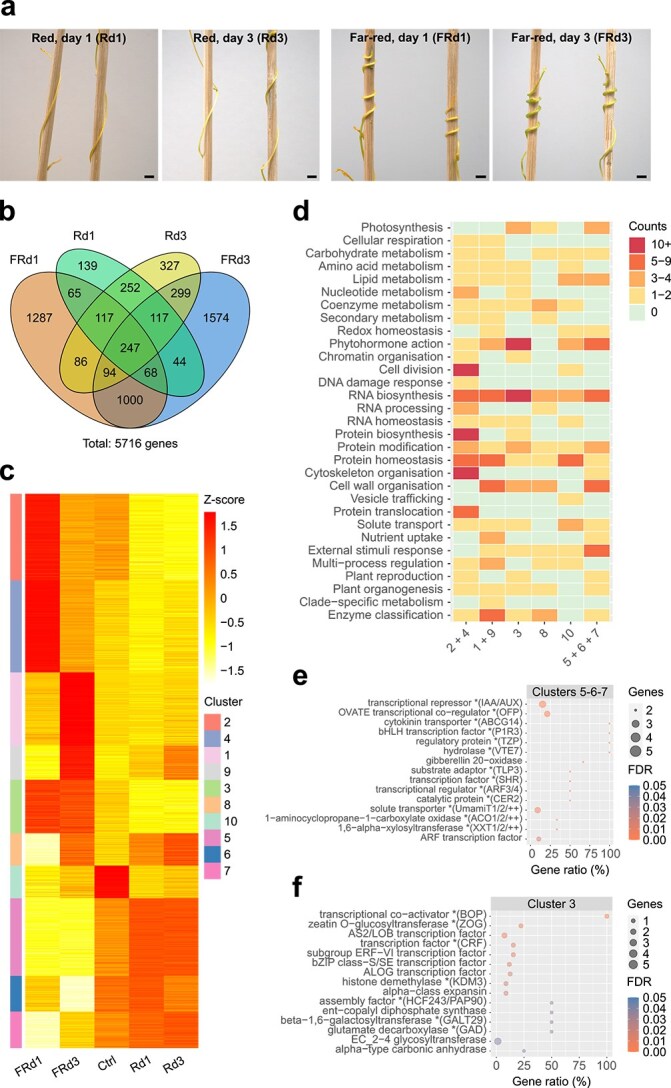
Gene expression dynamics in response to (far-)red light. (a) Phenotype of *C. campestris* shoots, each given a 3-mm-thick wooden stick as surrogate host after exposure to R- or FR-enriched light conditions for 1 and 3 days, respectively. The scale bar is 3 mm. (b) Representation of Venn intersections relative to natural light (Ctrl). Numbers represent DEGs. Only genes that showed an FDR-corrected *P*-value ≤ 0.05 and a |log_2_FC| ≥ 1.5 were retained. (c) Soft clusters of DEGs as a function of light. Values are average *z*-scores obtained from TPM counts. A *z*-score value is positive (negative) if the gene expression in a sample type is larger (smaller) than the overall mean expression. Only genes with a cluster membership ≥ 0.7 were retained. (d) Schematic representation of MapMan4 v. 7.0 enrichment in functional categories as a function of clusters. Counts refer to the number of bins that are significantly enriched with genes inside each top-level category. Redundant clusters (with an overall similar pattern of expression) were grouped together as supported by a correlation test. (e) Top enriched functional terms in clusters 5–7 (with genes downregulated by FR light at Days 1 and 3). ‘Gene ratio’ refers to the number of genes found in these clusters with a term relative to the number of genes in the transcriptome with that term. (f) Same as (e) with cluster 3 (with genes upregulated by FR light at Days 1 and 3).

The inert surrogate hosts did not alter previously observed phenotypic responses: in red-light-enriched conditions, the parasite wound only loosely around the wooden sticks and the shoots remained entirely devoid of haustoria, while in FR-light-enriched conditions tight coiling and swelling, indicating haustorium initiation (Bawin et al. 2022), was already observed within 24 h (Fig. 1a). After 3 days, the swelling was clearly developed. After 5 days, the coils were already too firmly attached to the sticks to be harvested without damaging, so only the middle of the monochromatic period (see Supplementary Data S1) on Day 1 and Day 3 was used as sampling time points for RNAseq. Shoots not exposed to either of the two light treatments (corresponding to Day 0) were used as reference samples. Differential expression analysis between (far-)red-light-treated shoots and untreated shoots harvested under natural light revealed that roughly three-quarters (75%) of regulated genes (3861 out of 5716) were up- or downregulated in the presence of FR light (Fig. 1b, Supplementary Fig. S2, and Supplementary Table S1). Transcriptional dynamics were then examined using a soft-clustering approach, which grouped the differentially expressed genes (DEGs) into 10 clusters with similar expression profiles (Fig. 1c and d, Supplementary Fig. S3, and Supplementary Data S2). Three of these comprised genes that were specifically downregulated by FR light at Days 1 and 3. These included *CcPHYB1b* (Cc031752) and *PIF* genes along with relevant enriched regulatory genes encoding for instance Auxin/Indole-3-Acetic Acid (AUX/IAA) transcriptional regulators, Auxin Response Factors (ARFs), and TANDEM ZINC-FINGER/PLUS3 (TZP) regulatory proteins (Fig. 1e), possibly sustaining cell elongation and cell division during the rapid shoot elongation that is observed under red light. *CcPHYB1a* (Cc031342) did not pass the three-fold change cut-off in expression to be included in the analysis. Five clusters involved genes that were specifically upregulated by FR light at Day 1, Day 3, or both. These included *CcPHYB2a* (Cc020059) and *CcPHYB2b* (Cc044589) (Yokoyama et al. 2025) along with many enriched transcription factors and other regulatory genes (Fig. 1f), consistent with the profound morphological changes that are observed during haustorium initiation and expansion. No gene identified as PIF-encoding by MapMan4 was upregulated under FR light and clustered with the *CcPHYB2* genes. It should however be noted that a gene encoding for a FAR-RED ELONGATED HYPOCOTYL 1/FHY1-LIKE (Fhy1/Fhl) regulatory protein of PhyA translocation was found as well in these upregulated clusters (Supplementary Data S2), suggesting the possible involvement of multiple phytochrome-related pathways in triggering transcriptional responses to FR light even in the absence of *PHYA*-gene regulation itself. The expression of selected phytochrome genes was validated by quantitative real-time reverse transcription PCR (qRT-PCR). To reveal whether the expression changes underlie faster dynamics that were not captured by the time points selected for RNAseq, sampling time points before, during, and after the 2-h R or FR period were included in this analysis (Supplementary Fig. S4 and Supplementary Table S2). This not only confirmed the opposite regulation of *CcPHYB2* compared to *CcPHYB1* but indicated constant levels without strong fluctuations. Together, these eight clusters of regulated genes (thereafter referred to as ‘FR-DEGs’) showed a clear signature of cellular growth processes, emphasizing the role of FR light signalling in dynamically modulating photomorphogenic development in *C. campestris* shoots.

### Far-red light triggers the expression of genes involved in epigenetic modifications

**Figure 2:**
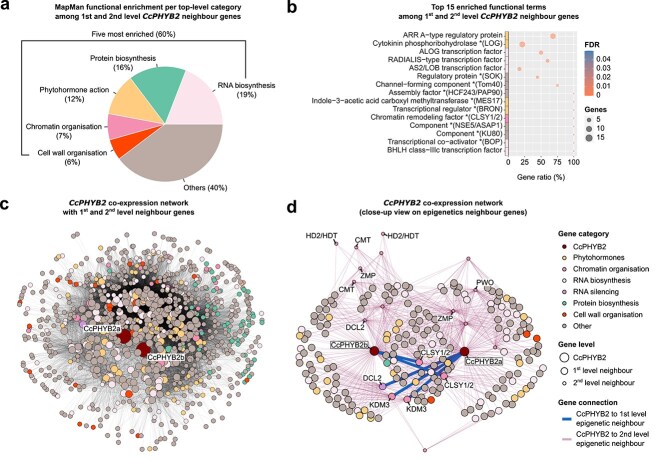
Phytochrome B2 coexpression network. (a) Schematic representation of enriched MapMan4 v. 7.0 functional terms per top-level category within the phytochrome B2 (Cc020059 and Cc044589) coexpression network (first- and second-level neighbour genes). (b) Top enriched functional terms. ‘Gene ratio’ refers to the number of genes with a term relative to the number of genes in the transcriptome with that term. (c) Phytochrome B2 coexpression network, showing the close connection of genes involved in phytohormone signalling and RNA biosynthesis (first level), along with genes involved in chromatin organization (including epigenetic modifications) and RNA silencing. Only the shortest paths to phytochromes are displayed. The size of the nodes (genes) is proportional to their neighbourhood level. (d) Close-up view of the network, showing first- and second-level connections between phytochromes B2 and genes involved in chromatin organization and RNA silencing. Genes with an expected epigenetic role are labelled. *PHYB2* = phytochrome B2; *CLSY* = RDR2-polymerase CLASSY accessory protein; *CMT* = DNA chromomethylase; *DCL* = Dicer-like protein; *HD2/HDT* = HD2-type histone deacetylase; *KDM* = histone demethylase; *PWO* = histone H3 methylation reader; *ZMP* = ‘Zinc finger, Mouse double-minute/switching complex B, Plus-3 protein’.

To further narrow down the FR-DEGs to genes that are in close association with *CcPHYB1b* and both *CcPHYB2* genes, we constructed a gene co-expression network (GCN) for both the down- and upregulated FR-DEGs [Pearson correlation coefficient (PCC) cut-offs of 0.98 and 0.97, respectively, with 6.53% and 6.07% of retained edges] and retrieved the first- and second-level connections. This confirmed the prevalent connection of *CcPHYB1b* to genes involved in external stimuli perception, phytohormone signalling, RNA biosynthesis, and cell wall organization, including a *PIF* gene and the other regulatory genes mentioned in the above section (Supplementary Fig. S5 and Supplementary Data S3). It also confirmed that *CcPHYB2* and *FHY1/FHL* genes had a strong regulatory connection, and that both were strongly connected to a large panel of genes involved in signalling and biosynthetic processes (Fig. 2a–c and Supplementary Data S4), including a wide array of genes involved in cytokinin biosynthesis and perception together with several types of transcription factors known to trigger the development of lateral organs (Fig. 2b). Our GCN analysis further revealed a close connection between *CcPHYB2* and *FHY1/FHL* genes and several genes involved in chromatin modification and modulation of transcriptional activity, including a histone methylation reader, demethylases, and deacetylases along with chaperones (Fig. 2d). Other such connected genes also included two components of the *de novo* RdDM pathway with CLASSY (*CLSY*) chromatin remodellers and RNA Pol IV regulators, together with chromomethylases putatively involved in RNA-independent maintenance of DNA methylation. In line with this, *DCL2* genes for endoribonucleases involved in sRNA biogenesis were also found. Four of the six miRNA-degrading Small RNA Degrading Nucleases (SDNs) identified by MapMan4 were consistently and significantly upregulated as well at Day 1. Together, these observations support that epigenetic regulation involving histone modifications, sRNA-mediated silencing, and DNA methylation plays a role in controlling haustorium development downstream of phytochrome-mediated perception of FR light.

### sRNA profiling suggests posttranscriptional gene regulation via miRNAs in response to light

**Figure 3:**
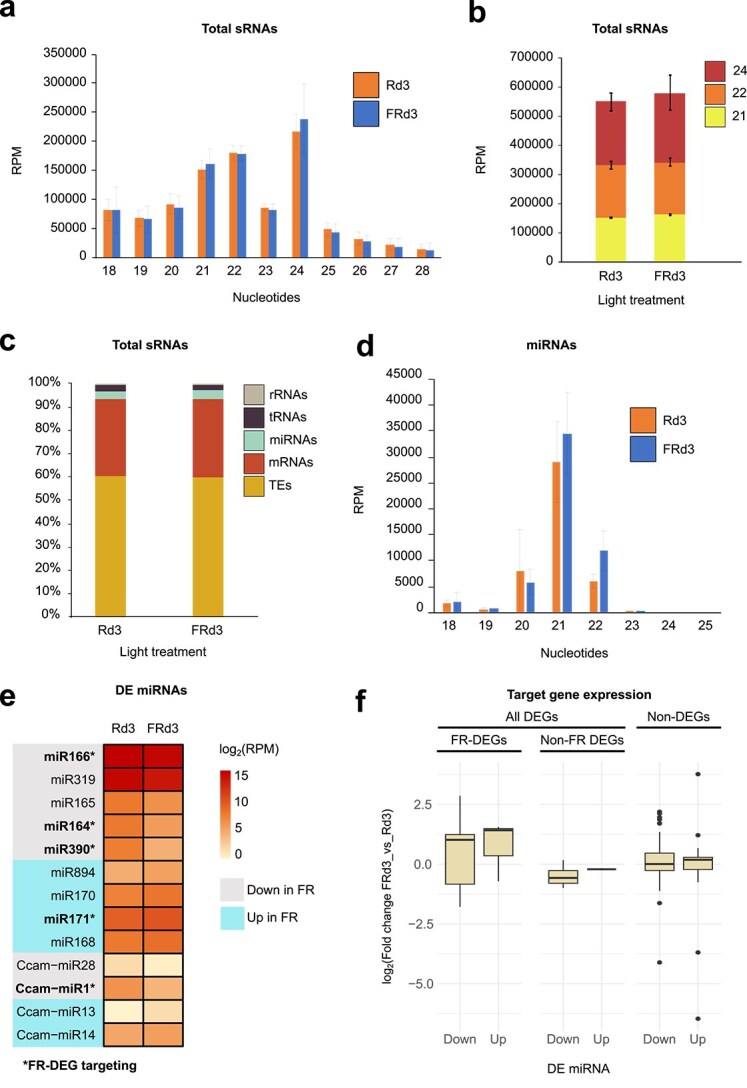
Profiling of sRNAs and miRNA target gene expression. (a, b) Average total sRNA abundance per size class and light condition. (c) Categorization of total sRNAs between light conditions. (d) Average miRNA abundance per size class and light condition. (e) Differentially expressed miRNA families in response to light, including FR-DEG-targeting families. (f) Expression shifts of confirmed and predicted FR-DEG target genes of those differentially expressed FR-DEG-targeting miRNAs.

To investigate sRNA dynamics in *C. campestris* under different light conditions, we generated sRNA libraries of the same RNA pools at Day 3 that were used for the mRNA analysis (Supplementary Table S3). The overall sRNA size distribution (18–28 nt) remained consistent across treatments, with 21-, 22-, and especially 24-nt sRNAs being the most abundant (Fig. 3a and b). Most sRNAs were derived from transposable elements (TEs, ~60%) and mRNAs (~33.5%), followed by microRNAs (miRNAs, ~3.5%), transfer RNAs (tRNAs, ~2.5%), and ribosomal RNAs (rRNAs, ~0.5%), with no significant differences in overall accumulation or size distribution between light conditions for any category (Fig. 3c). Despite this, we observed that nine conserved and four species-specific miRNA families were affected by the light treatments (Fig. 3d and e). Of these, four conserved miRNA families and one species-specific miRNA family targeted ~0.45% (19 of 4229) of the FR-DEGs. These included upregulation of the miR171 family and downregulation of the miR166, miR164, and miR390 families, and of our previously identified species-specific Ccam-miR1 (Zangishei et al. 2022) (Fig. 3e and Supplementary Table S4). To understand their potential role, we analysed the expression values of their targeted genes. We found that a GRAS transcription factor gene (*Cc034532*) was consistently downregulated, while its putatively controlling miR171 was upregulated. By contrast, 11 of the 19 FR-DEG targets showed a significant increase in their expression under FR light in line with the downregulation of the miRNAs possibly controlling them (Fig. 3f and Supplementary Data S5). This included for instance a plant-specific organogenesis gene (*Cc002635*) involved in nonparasitic plants in leaf abaxial/adaxial polarity that is targeted by miR166. These findings suggest that while the overall sRNA profile in *C. campestris* remained stable across light treatments, the limited number and overlap of differentially expressed miRNAs with FR-DEGs could play a role in modulating development in response to light, pointing to alternative and overlapping regulatory pathways.

### Differential DNA methylation affects far-red-light-regulated genes

**Figure 4:**
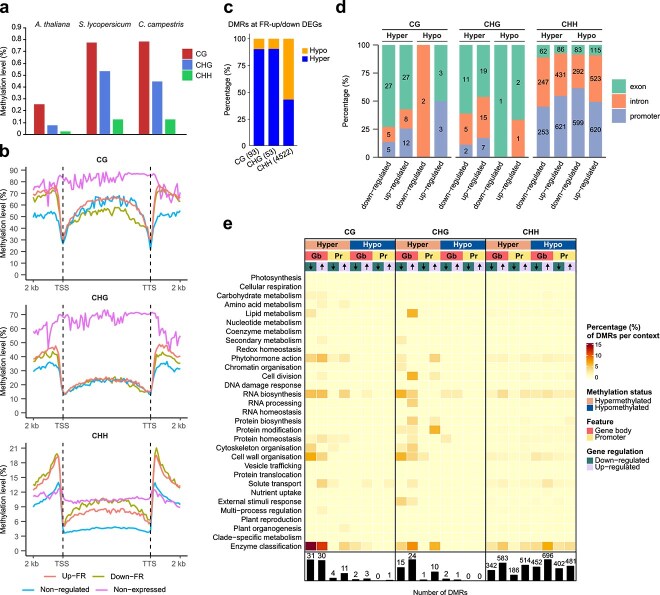
DNA methylation changes induced by FR light. (a) Global average DNA methylation levels of CG, CHG, and CHH contexts in *A. thaliana*, *S. lycopersicum*, and *C. campestris*. (b) DNA methylation profile in protein-coding genes from transcription starting site (TSS) to termination site (TTS). The length of each gene and its 2-kb flaking regions was normalized and divided into equal numbers of bins. Values are mean cytosine methylation levels per bin. (c) Proportion (%) of hypermethylated (i.e. with higher methylation level under FR than R light) and hypomethylated DMRs per context in the promoter and gene body of FR-DEGs. (d) Categorization per methylation context of hyper- and hypomethylated DMRs that are associated with FR-DEGs, as a function of genomic features. A DMR may be linked to multiple genomic features. DMRs located in annotation gaps are not included. (e) Schematic representation of DMRs associated with FR-DEGs as a function of MapMan4 v. 7.0 functional categories. Colours in the heatmap refer to the percentage (%) of DMRs per methylation context (CG, CHG, or CHH) inside each top-level category.

To investigate the relationship between DNA methylation and light-dependent changes in gene expression, we assessed the profile of methylated cytosines in the *Cuscuta* epigenome after 3 days of exposure to the different light regimes using bisulphite sequencing (Supplementary Table S5). Despite their different morphologies and lifestyles, interspecies comparisons showed that overall DNA methylation levels in *C. campestris* were similar to those from the crop species *Solanum lycopersicum* from the same order (Solanales) (Fig. 4a). Global methylation profiles in protein-coding genes and TEs were consistent across both light conditions (Supplementary Fig. S6a–c). However, methylation levels substantially increased in all methylation contexts in the 2 kb up- and downstream genomic regions of genes that were differentially regulated in the two light regimes compared to genes that showed constitutive expression. Methylation levels in the CHH context also increased in the body of light-regulated genes relative to nonregulated genes (Fig. 4b). To identify regions in the genome harbouring changes in methylation upon light treatments, we identified differentially methylated regions (DMRs). Our analysis revealed a total of 55 308 DMRs in response to light across the *C. campestris* genome (Supplementary Fig. S6d–g). Many of the DMRs in genic and TE regions were associated with protein-coding genes (58% of 33 851) or with both protein-coding genes and TEs (18%), rather than with TEs alone (24%) (Supplementary Fig. S6h and i). Approximately 56% (2354 of 4229) of the FR-DEGs possessed DMRs, covering ~8% of the total identified DMR population (Fig. 4c and d). Of these DMRs the CHH context (~97%) was the most strongly represented compared to the CG and CHG contexts (2% and 1%). Interestingly, while CG and CHG DMRs were mostly associated with hypermethylation (~90%), CHH DMRs showed more variation, with 55% and 45% associated with hypo- and hypermethylation, respectively (Fig. 4c). Of the DMRs that were assigned to gene features in FR-DEGs, CG and CHG DMRs were predominantly associated with exons (62% and 53%) rather than promoter regions (22% and 14%) and introns (16% and 33%), whereas CHH DMRs were strongly connected to introns (38%) and promoter regions (53%) rather than exons (9%) (Fig. 4d). The top five represented MapMan functional categories in DMR-targeted FR-DEGs were enzyme classification, RNA biosynthesis, solute transport, cell wall organization, and phytohormone action (Fig. 4e).

### Far-red light may shape gene expression through diverse epigenetic interactions involving DNA methylation

**Figure 5:**
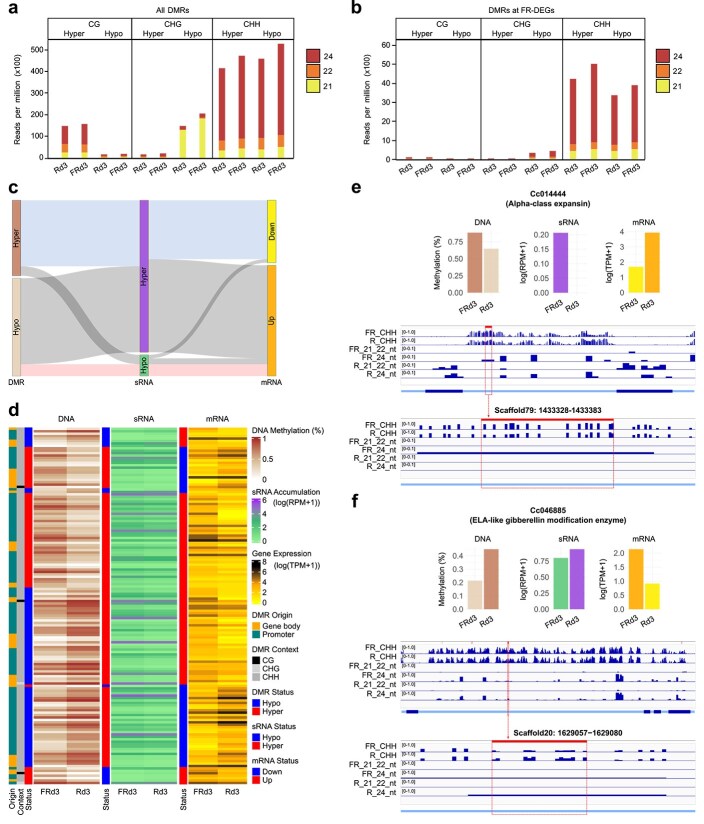
Multiomics analysis of gene regulation under (far-)red light. (a) Average abundance of 21-, 22-, and 24-nt sRNAs at hyper- and hypomethylated DMRs under R and FR lights considering all the DMRs. (b) Same as (a) considering DMRs at FR-DEGs. (c) Sankey diagram of genes that show differential DNA methylation, 21-, 22-, and 24-nt sRNA accumulation, and regulation of their expression under (far-)red light. The size of the nodes and links is proportional to their frequency. ‘Hyper’ (‘Hypo’) and ‘Up’ (‘Down’) refer to an increase (decrease) of the measured parameter under FR light. (d) Heatmap visualization of the multiomics dataset presented in (c), with each row being a DMR in an FR-DEG where sRNAs differentially accumulate. (e) Gene expression of *Cc014444* after 71-h exposure to light and corresponding (CHH) DNA methylation and sRNA accumulation in its only predicted DMR, with Integrative Genomics Viewer (IGV) visualization. The DMR region is represented on top of the tracks and delineated with dotted lines. Genomic features including introns and exons (thickened sections) are represented below the tracks. (f) Same as (e) with *Cc046885* and its four predicted DMRs. The one DMR with differential accumulation of sRNAs is emphasized.

Our transcriptomic data suggested that the two *CcPHYB2* genes may be linked to changes in the expression of key components of the RdDM pathway such as *CLSY* and *PolIV*, prompting us to further explore the connection between DNA methylation and sRNAs in FR-light-regulated gene expression. The overall analysis of sRNA presence at DMRs indicated that sRNAs were predominantly associated with CHH DMRs in both hyper- and hypomethylation, and with hypermethylated DMRs in the CG context and hypomethylated DMRs in the CHG context, indicating their potential connection with RdDM activity (Fig. 5a). Despite this, no obvious difference in sRNA accumulation was observed when considering only CG DMRs at FR-DEGs (Fig. 5b). Similarly, when considering all the DMRs, a relatively higher abundance of 21-, 22-, and 24-nt sRNAs was observed in hypomethylated CHG DMRs compared to their hypermethylated counterparts, with a clear predominance of 21-nt sRNAs. No such difference was observed when considering only CHG DMRs at FR-DEGs. Rather, a slight predominance of 24-nt sRNAs was observed. CHH DMRs were associated with a higher abundance of 21-, 22-, and especially 24-nt sRNAs, regardless of differential expression in protein-coding genes. By contrast, the accumulation of 21-, 22-, and 24-nt sRNAs in TEs was consistent between light conditions (Supplementary Fig. S7). Only 3.1% (147 of 4668) of the predicted DMRs in FR-DEGs showed significant differential accumulation of sRNAs at 71 h light exposure. Most of these DMRs, regardless of hyper- or hypomethylation, were in the CHH context in the gene promoter region, and they were largely associated with statistically significant 21-, 22-, and 24-nt sRNA hyperaccumulation, which in turn was associated to a greater extent with gene upregulation (Fig. 5c and d). These observed patterns indeed suggest a diverse and dynamic epigenetic landscape where DNA methylation, sRNAs, and gene expression might be interconnected. Under the hypothesis that the RdDM pathway controls these DMRs, it is expected that increased levels of DNA methylation positively correlate with sRNA accumulation, while they correlate negatively with gene expression (i.e. downregulated mRNA levels), and vice versa. Out of the subset of 147 predicted DMRs mentioned above, 29 (19.7%) exhibited such behaviour. More than half of these (19 genes) were downregulated genes possessing hypermethylated DMRs (Supplementary Data S6). Interestingly, among the handful of genes that showed consistent patterns of DNA methylation and sRNA accumulation matching the above-mentioned pattern of expression, four were members of the *CcPHYB1b* network (Supplementary Fig. S5 and Supplementary Data S3) that are downregulated in FR-enriched conditions, one of them playing a role in cell wall organization (*Cc014444*) (Fig. 5e), another being a MASS regulatory protein of stomatal patterning (*Cc018741*), and the others not being classified by MapMan4 (*Cc015566* and *Cc044206*). Three members of the *CcPHYB2* network, in contrast, where increased expression under FR light correlated with hypomethylation and with depletion of sRNAs, are involved in RNA biosynthesis (*Cc045554*) and phytohormone action (*Cc026122* and *Cc046885*) (Fig. 5f). Altogether, those genes are specific instances of a possible regulation of their expression downstream of phytochrome signalling that can be driven by the RdDM pathway. It should however be noted that several of these RdDM candidate genes may have additional DMRs of the same type in their promoter or gene body regions that lack apparent connections to a significant differential sRNA accumulation (Fig. 5f), stressing that DNA methylation–regulating mechanisms other than RdDM are likely to also contribute to shaping the methylome.

## Discussion

The current model of light perception in *Cuscuta* implies that phytochromes switch towards their inactive (P_R_) form under FR light, thus unrestricting their interaction partners from regulation, which in turn activates the genes that are required to initiate haustorium development (Furuhashi et al. 2011, Jhu and Sinha 2022). Our data corroborated the work of Yokoyama et al. (2025) with diametrically contrasting patterns of expression for *CcPHYB1* and *CcPHYB2*, suggesting diverging functions in regulating responses to light. The increase in expression of *CcPHYB1* genes under R light, and decrease under FR light, together with a higher level of sequence conservation, point to an activity for these genes that is similar to that of *AtPHYB.* This observation converges to a model of light signalling in which some master regulator downstream of phytochromes evolved a different function in *Cuscuta*, leading to haustorium development. In this context, it should be noted that some downstream regulators like *HMR* (*HEMERA*) (Galvão et al. 2012), which mediate the degradation of *PHYB*-antagonistic PIFs, were lost from the genome of *C. campestris*, exemplifying the differences that exist between the parasite and canonical models of phytochrome signalling in fully photosynthetic plants.

The completely opposite pattern of expression of *CcPHYB2* genes, on the other hand, could suggest that these phytochromes accumulate in greater amounts under low R:FR ratios. However, it would be counterintuitive if they would at the same time be converted to their inactive form, as this would question the biological relevance of such an increase. The expression pattern would make more sense under the assumption that the P_R_ form of these phytochromes in *Cuscuta* is the active form. Despite long-standing general assumptions, this possibility is supported by recent experimental evidence from *A. thaliana* (Li and Hiltbrunner 2021). The observation that *HY5* and most *PIF* genes are transcriptionally associated with red light does not exclude a role in parasitism for these, but suggests that *CcPHYB2* may interact with other, as yet unidentified, partners that exert a predominant regulatory influence. Although not regulated by light in our study, phytochrome A was previously found in *A. thaliana* to be a positive regulator of photomorphogenesis under FR light, thus being important for seedling establishment in strong canopy shade (Menon et al. 2020, Cheng et al. 2021). *FHY1* mediates key steps of AtPhyA signalling, including its nuclear translocation, interaction with transcription factors, and promoter binding (Cheng et al. 2021). The regulation of a *FHY1/FHL* gene, which was here highly coexpressed with *CcPHYB2* genes, may indicate the modulation of the activity of CcPhyA proteins, even if the expression of *CcPHYA* genes is not regulated. It should be stressed that *FHY1* is also known to have an independent role in gene modulation and plant development under FR light (Chen et al. 2014). Our results therefore support the possible interaction between multiple light signalling pathways to regulate haustorium development downstream of FR light perception.

Our data further confirmed the considerable contribution of hormones and transcription factors presumably involved in lateral organ development and organization to haustorium initiation, with a massive representation of cytokinin-related genes in the *CcPHYB2* coexpression network, and a high prevalence of auxin-related genes showing coregulation with *CcPHYB1* genes. The early increase in cytokinin in response to the FR signal is indeed a long-established feature of haustorium development in *Cuscuta* and a requirement for proper coiling and development of a functional parasitic organ (Tsivion 1978, Gupta and Singh 1985, Rajagopal et al. 1988, Ramasubramanian et al. 1988, Haidar et al. 1998, Furuhashi et al. 2021, Pan et al. 2022). Cytokinin interacts with low auxin as a prelude to haustorium formation, which is otherwise inhibited by high auxin; the effect of these hormones is light-dependent and suggested to be under the control of phytochromes (Rajagopal et al. 1988, Haidar et al. 1998, Furuhashi et al. 2021). Evidence now suggests that hormone action and epigenetic mechanisms in *A. thaliana* are interdependent: hormone biosynthesis, transport, and signalling may be modulated by sRNAs and epigenetic factors, and hormones may as well modulate the epigenetic landscape (Yamamuro et al. 2016, Torres et al. 2019, Wang et al. 2022). This is consistent with our observations in *Cuscuta*. In addition to hormones, an apparent close connection between *CcPHYB2* and *FHY1/FHL* genes, and genes involved in histone modifications, RNA-(in)dependent DNA methylation and RNA silencing support a strong role of epigenetic reprogramming during photomorphogenetic haustorium development in the parasite, and tentatively interconnect hormonal, phytochrome, and epigenetic control pathways.

Our data clearly showed that the light treatments modified the sRNAome and DNA methylome profiles, which correlated with many transcriptomic alterations. This indicates that phytochromes in *Cuscuta*, indeed, regulate these processes at least in part, through both transcriptional and posttranscriptional gene silencing mechanisms. Although the overall sRNA profile in *C. campestris* remained largely unchanged across light treatments, the small number and limited overlap of differentially expressed miRNAs with FR-responsive genes suggest that miRNAs may have a targeted role in modulating development in response to FR light. The upregulation of miRNA-degrading SDN exoribonucleases at Day 1 under enriched FR light may have contributed to the observed predominant downregulation of miRNAs at Day 3. An interesting target (*Cc002635*) of these miRNAs is a homologue of AT5G60690 (REVOLUTA) in *A. thaliana*, which regulates lateral meristem initiation (Liu et al. 2024). Further, the coordinated expression of genes from distinct epigenetic pathways under FR-enriched light, along with the contrasting DNA methylation patterns across CG/CHG and CHH contexts, suggests that DNA methylation modulates gene expression through multiple, context-dependent mechanisms. Especially, the upregulation of histone modifiers and genes involved in the establishment and maintenance of DNA methylation and the limited number of genes that showed consistent patterns of methylation and sRNA accumulation in relation to their pattern of expression converge to a preponderant role of histone marks and DNA methylation in modulating chromatin accessibility and subsequent transcriptomic responses (Zhang et al. 2018). Our data support a role of the RdDM pathway in regulating the expression of specific genes downstream of light signalling. *CLSY* genes, which were here found in close connection with *CcPHYB2* and *FHY1/FHL* genes, are an example of well-known regulators of tissue-specific DNA methylation (Long et al. 2021, Zhou et al. 2022). Examples of RdDM-regulated genes that we identified in our study include two members of the *CcPHYB2* network (*Cc026122* and *Cc046885*) that are involved in hormone degradation. Silencing of the phytochrome-controlled gene *AT1G75450* in *A. thaliana*, which is a homologue of one of them, results in larger plants and increased biomass (Zhang et al. 2011, Nomoto et al. 2012). Similarly, *AT1G15400*, being homologous to a putative MAPK kinase substrate protein (*Cc018741*) downstream of *CcPHYB1b*, is known to regulate stomatal production and patterning, with mutant plants having a lowered stomatal index (Xue et al. 2020). While *Cuscuta* only has a few stomata at best (Clayson et al. 2014), an increase in the expression of such genes under red light is most likely not connected to stomata function, even though photosynthetic activity may increase in foraging *Cuscuta* shoots as a survival strategy, as suggested by the modulation of chlorophyll synthesis in the absence of a host (Parise et al. 2021). It is important to note that several of the identified RdDM candidate genes contained additional DMRs of the same type in their promoter or gene body regions that did not show a clear association with differential sRNA accumulation. This highlights the likely involvement of non-RdDM DNA methylation pathways in shaping the methylome and raises questions about the biological significance and interdependence of the identified DMRs. Functional genomic studies are now required to further elucidate causal connections between these epigenetic processes and regulation of gene expression downstream of light perception in *Cuscuta*, and their significance for the development of infective structures. Although *Cuscuta* species have traditionally been notoriously difficult to genetically transform, recent and ongoing advances in transformation and regeneration protocols offer promising new avenues (Lachner et al. 2020, Adhikari et al. 2025, Alles et al. 2025).

## Materials and Methods

### Plant materials

Plants were maintained at the Climate Laboratory of the Arctic University of Norway, Tromsø, in a greenhouse under 24 h light and ~21°C. *Cuscuta campestris* was originally obtained from the Botanical Garden of the University of Kiel (Germany) and propagated using *Pelargonium zonale* as a host.

### Light treatments

Harvested shoot tips of *C. campestris* (exactly 17.5 cm in length) from its *P. zonale* host were attached to a wooden stick by loosely winding each shoot one or two turns around its own surrogate ‘host’. With the cut side submerged in an Eppendorf tube with water, they were placed under Heliospectra RX30 lamps using one of two specific LED settings, unless otherwise specified: for obtaining red-enriched conditions 6 h of darkness were followed by 16 h of red-supplemented white light (~330 *μ*mol/m^2^/s) and 2 h of red LED light (~30 *μ*mol/m^2^/s) to conclude one 24-h cycle. For obtaining FR-enriched conditions, the same schedule was used but with FR-supplemented white light (~350 *μ*mol/m^2^/s), followed by 2 h of pure FR LED light (~30 *μ*mol/m^2^/s). The spectra and intensities of each treatment are shown in Supplementary Fig. S1. Shoots harvested before the transfer to R or FR light conditions (*t* = 0) served as reference for the FR- or R-light time series that were harvested after 21, 23, 25, 69, 71 and 73 h. All six time points were analysed by qRT-PCR, while only the sampling in the middle of the monochromatic (far-)red period after 23 (1-day R or FR samples) or 71 h (3-day R or FR samples), respectively, were used for mRNAseq and small RNAseq. DNA methylome sequencing was only done with the 3-day (71 h) samples. The apical 0.5 cm of the shoot was discarded, as was the distal ~12 cm, leaving a region of 5+ cm where *Cuscuta* coiled and grew that was kept. The trimming was done with fresh razor blades under green light, and a minimum of three coiled shoot segments were pooled together before being frozen in liquid nitrogen. At least 20 shoot/wooden stick pairs were set up per condition and time point, allowing us to harvest four to six replicates consisting of three pooled shoots each for every analysis. Four replicates of each treatment and time point were selected for sequencing based on the obtained RNA integrity values.

### Extraction, library preparation, and sequencing

Total RNA extraction was done as described in Bawin et al. (2023) using the Maxwell 16 low-elution-volume Plant RNA Kit and the Maxwell 16 Instrument (Promega). The quality and quantity of the RNAs were determined using a combination of NanoDrop (Thermo Fisher Scientific), Qubit (Thermo Fisher Scientific), and Experion (Bio-Rad). DNA was extracted using the E.Z.N.A. HP Plant and Fungal DNA Kit (Omega Bio-tek) including the optional addition of 10 *μ*L 2-mercaptoethanol and 2 *μ*L RNase A. DNA concentration was measured with a Qubit fluorometer, and quality was assessed using a NanoDrop spectrophotometer and gel electrophoresis. Library generation and sequencing of mRNAs (NovaSeq 6000 PE150, 9 G/sample) and sRNAs (NovaSeq 6000 SE150, 10 M per sample), and whole-genome bisulphite sequencing (NovaSeq 6000 PE150, 15 G per sample, 30× coverage), were outsourced to a commercial service provider (Novogene).

### Quantitative real-time reverse transcription PCR (qRT-PCR)

Gene-specific primer pairs were designed using Primer3 (v. 2.4.0) (Untergasser et al. 2012) and checked for target specificity using a locally run server version of MFEprimer3.1 (Wang et al. 2019) (Supplementary Table S2). cDNA synthesis from isolated RNA and real-time PCR were performed as described by Bawin et al. (2022). Average target abundances were based on technical duplicates and normalized to the untreated controls as well as to the housekeeping genes *RPT3* (*Cc001025*) and *SKIP/MAC6* (*Cc003338*).

### mRNA profiling

Quality control and read trimming were performed using fastp v. 0.21.0 (Chen 2023) with default parameters. Mapping on the *C. campestris* genome (Vogel et al. 2018) was performed using hisat2 v. 2.1.0 (Kim et al. 2019) with default parameters. Mapped read pairs were counted on the gene models using HTSeq v. 0.11.2 (Putri et al. 2022). Counts were normalized into transcripts per kilobase million (TPM).

### Differential gene expression analysis

DEGs were identified between sample groups using DESeq2 v. 1.34.0 (Love et al. 2014) with raw read counts as input data. *P*-values were adjusted using the Benjamini–Hochberg (BH) procedure. A false discovery rate (FDR) cut-off of 0.05 and a minimum log_2_ fold change [log_2_(FC)] of 1.5 were used to retain genes of interest.

### Soft gene clustering

The fuzzy *c*-means algorithm as implemented in Mfuzz v. 2.54.0 (Futschik and Carlisle 2005) was used to cluster, based on their log_2_(TPM) values, genes that (i) were differentially expressed in at least one of any possible pairwise comparison of light treatments and (ii) had a minimum of five normalized counts in at least three samples regardless of the treatment.

### Gene co-expression network

The PCC between expression profiles was calculated separately for genes upregulated (clusters 1–4 and 9) and downregulated (clusters 5–7) under FR light. PCC values were normalized by applying Fisher’s *Z* transformation and used to create an adjacency matrix with a normal quantile cut-off of 0.945. Coexpression networks were built and queried using igraph v. 1.3.5 (Csardi and Nepusz 2006).

### Gene annotation and enrichment analysis

Assignment of *C. campestris* genes to MapMan4 v. 7.0 functional categories was performed using the online Mercator annotation tool (Schwacke et al. 2019). Gene set enrichment analyses were performed by applying a hypergeometric test. *P*-values were adjusted using the BH procedure. Bins with an FDR ≤ 0.05 were considered significantly enriched.

### sRNA profiling

Read sequences were preprocessed by performing adaptor trimming with Trim Galore (Krueger 2015) and filtering according to their length (removing reads < 16 nt) and quality (Phred score of 20). The remaining sequences were aligned to the *C. campestris* genome (v. 0.32) using bowtie (Langmead et al. 2009) with the parameters -t -v2, allowing up to two mismatches. The sRNA accumulation level was normalized by calculating reads per million of 18–28 nt genome-matching sRNAs. Categorization was performed by sequentially and individually aligning the sRNA libraries to different indexes corresponding to genes, miRNAs, TEs, rRNA, and tRNA.

### DNA methylome analysis

Quality control and read trimming were performed using fastp v. 0.21.0 (Chen 2023) with default parameters to remove low-quality bases and adapter sequences. Reads with >50% of bases with a Phred score < 20 were also removed. Trimmed reads were aligned to the *C. campestris* genome (v. 0.32) using BISMARK v. 0.23.1 (Krueger and Andrews 2011) with default scoring options and the maximum insert size set to 700 bp, and methylation levels were calculated from cytosine conversions as methylated Cs/(methylated Cs + unmethylated Cs). Methylation profiles across genomic features were analysed using ViewBS v. 0.1.11 (Huang et al. 2018), and DMRs were identified using metilene v. 0.2-8 (Jühling et al. 2016). Regions were differentially methylated when the number of Cs was >5, the methylation difference was >0.1, and the BH-corrected *P*-value was <0.05. Overlap between DMRs and genomic features with a minimum of 1 bp were identified with bedtools v. 2.30.0 (Quinlan and Hall 2010).

## Supplementary Material

Supplementray_materials_pcag043

Data_S1_pcag043

Data_S2_pcag043

Data_S3_pcag043

Data_S4_pcag043

Data_S5_pcag043

Data_S6_pcag043

## Data Availability

The generated raw reads from this article can be found in the NCBI Sequence Read Archive (SRA) under the BioProject number PRJNA1269595. Expression data are available upon reasonable request.
