# Supplementary material for: Phytochrome-mediated light perception in dodders drives haustorium development through epigenetic mechanisms
Source: Plant Cell Physiol. 2026 Apr 2;67(4):685–99. doi: 10.1093/pcp/pcag043 (PMC13192454; doi:10.1093/pcp/pcag043)
Supplement: Supplementray_materials_pcag043 [file supplementray_materials_pcag043.docx]

**Phytochrome-Mediated Light Perception In Dodders Drives Haustorium Development Through Epigenetic Mechanisms**

Thomas Bawin^1^*, Andreas Evenstad^1^, Alena Didriksen^1^, German Martinez^2^, Kirsten Krause^1^*

^1^ Department of Arctic and Marine Biology, UiT The Arctic University of Norway, Tromsø, Norway

^2^ Department of Plant Biology, Uppsala BioCenter, Swedish University of Agricultural Sciences and Linnean Center for Plant Biology, Uppsala, Sweden

* Address correspondence to:

Thomas Bawin: [thomas.bawin@uit.no](mailto:thomas.bawin@uit.no)

Kirsten Krause: [kirsten.krause@uit.no](mailto:kirsten.krause@uit.no)

**Supplementary Data**


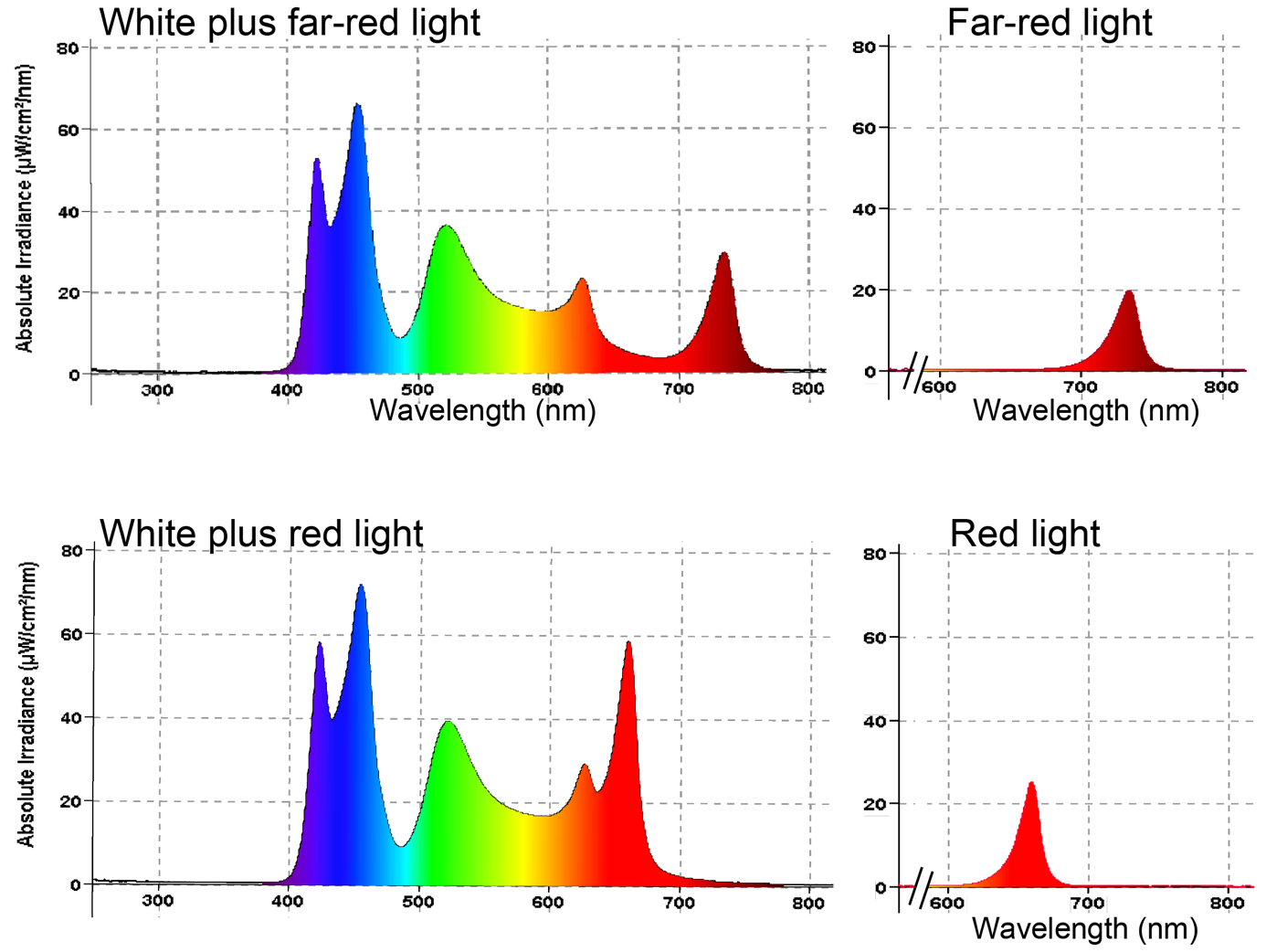


**Figure S1. Irradiance spectra of light treatments used in this study.** All experiments were done using LED-based Heliospectra RX30 lamps (Heliospectra AB, Gothenburg, Sweden). “Daylight” conditions were achieved by using 5300 K white light bulbs which were supplemented with either far-red (FR) light or red (R) light for the FR or R-treatments, respectively, resulting in the spectra shown on the top and bottom left. Additionally, monochromatic FR- or R-light (top and bottom right, respectively) was used for 2 hours following the 16 hours of “daylight” conditions and preceding a 6-hour night period. For controls, the FR and R light was omitted throughout the light/dark rhythm. Settings of the LEDs were: 1000 for 5300K bulbs, 300 for FR-bulbs and 100 for R bulbs, resulting in the irradiation energy fluxes (absolute irradiance, µW/cm^2^/nm) depicted in the graphs. The measurements of the latter were performed with a JAZ spectrometer (Ocean Optics Inc., Orlando, FL, USA).


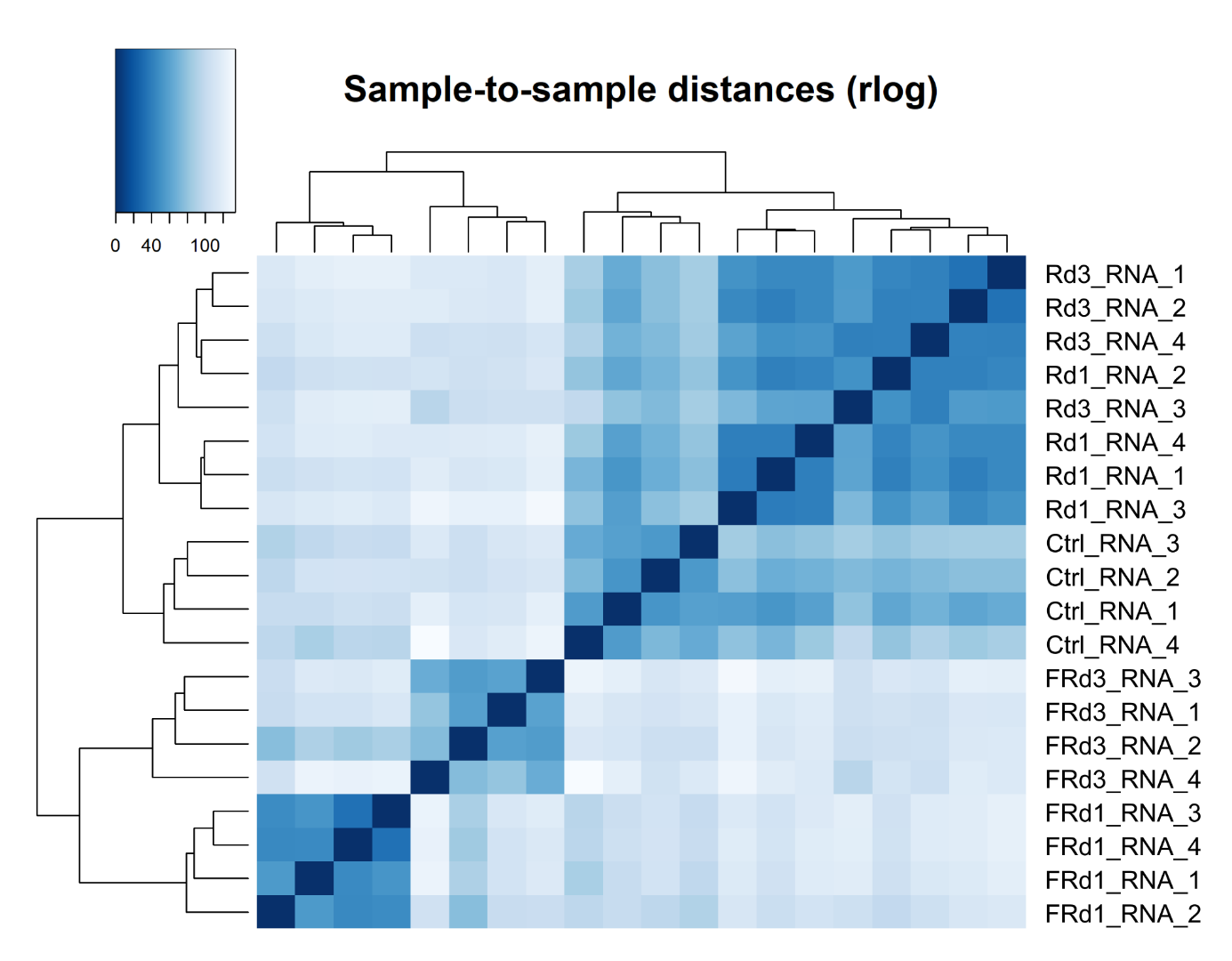


**Figure S2. Hierarchical clustering of sequenced biological replicates based on their transcriptomes.** Mapping of reads was performed on the *C. campestris* genome v. 032. Sample clustering is based on Euclidean distance between log_2_(counts-per-million + pseudocount) values. The map is symmetrical because the samples are plotted against each other. The darker the colour, the closer the samples. Ctrl = control, R = red light, FR = far-red light, d1 = day 1, d3 = day 3.


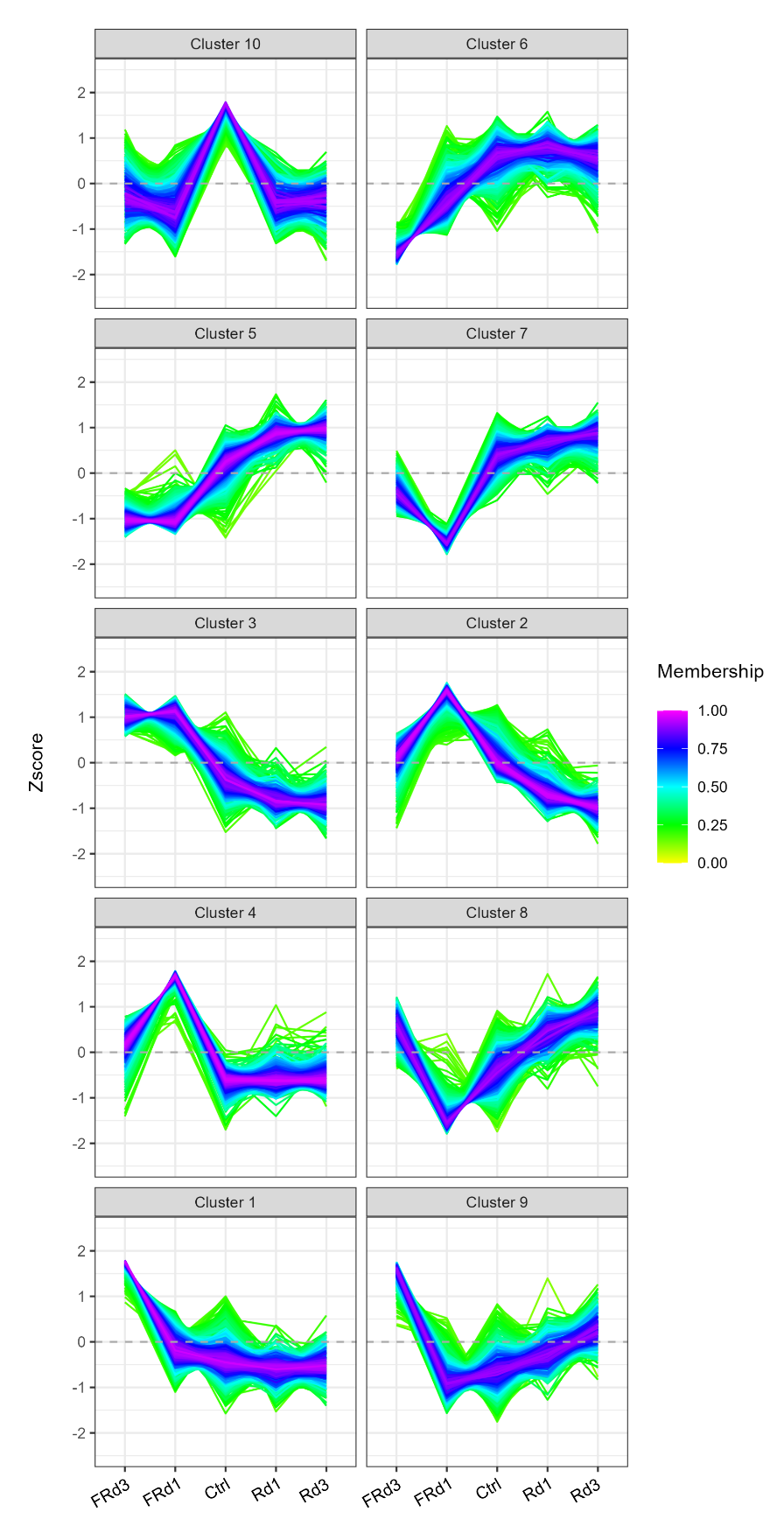


**Figure S3. Gene expression profiles in soft clusters.** Values are “Transcripts Per Kilobase Million” (TPM) values transformed into z-scores. A z-score value is positive (negative) if gene expression in a sample is larger (smaller) than the mean expression for that gene. Colours indicate the membership value (the higher the value, the better a gene fits into a cluster). Ctrl = control, R = red light, FR = far-red light, d1 = day 1, d3 = day 3.

**Figure S4. Heat map showing average log_2_ fold changes (FC) of genes for phytochrome A (a), phytochrome B1 (b), and phytochrome B2 (c).** Quantitative RT-qPCR values of the test genes were normalized against control samples and one of two house-keeping reference genes (2−ΔΔCT). The average and standard deviation values are based on 2 technical replicates of 3 biological replicates each. In addition to the same time points used for RNAseq (in the middle of the (far-)red light period (“(F)R”), two additional time points were taken two hours earlier (at the end of the white light period, (“W”)) and two hours later (at the beginning of the dark period, (“D”)). The heat map colours indicate proportional upregulation (>1, blue) or down-regulation (<1red). Standard deviation values (StDEV) are shown without colour coding below.


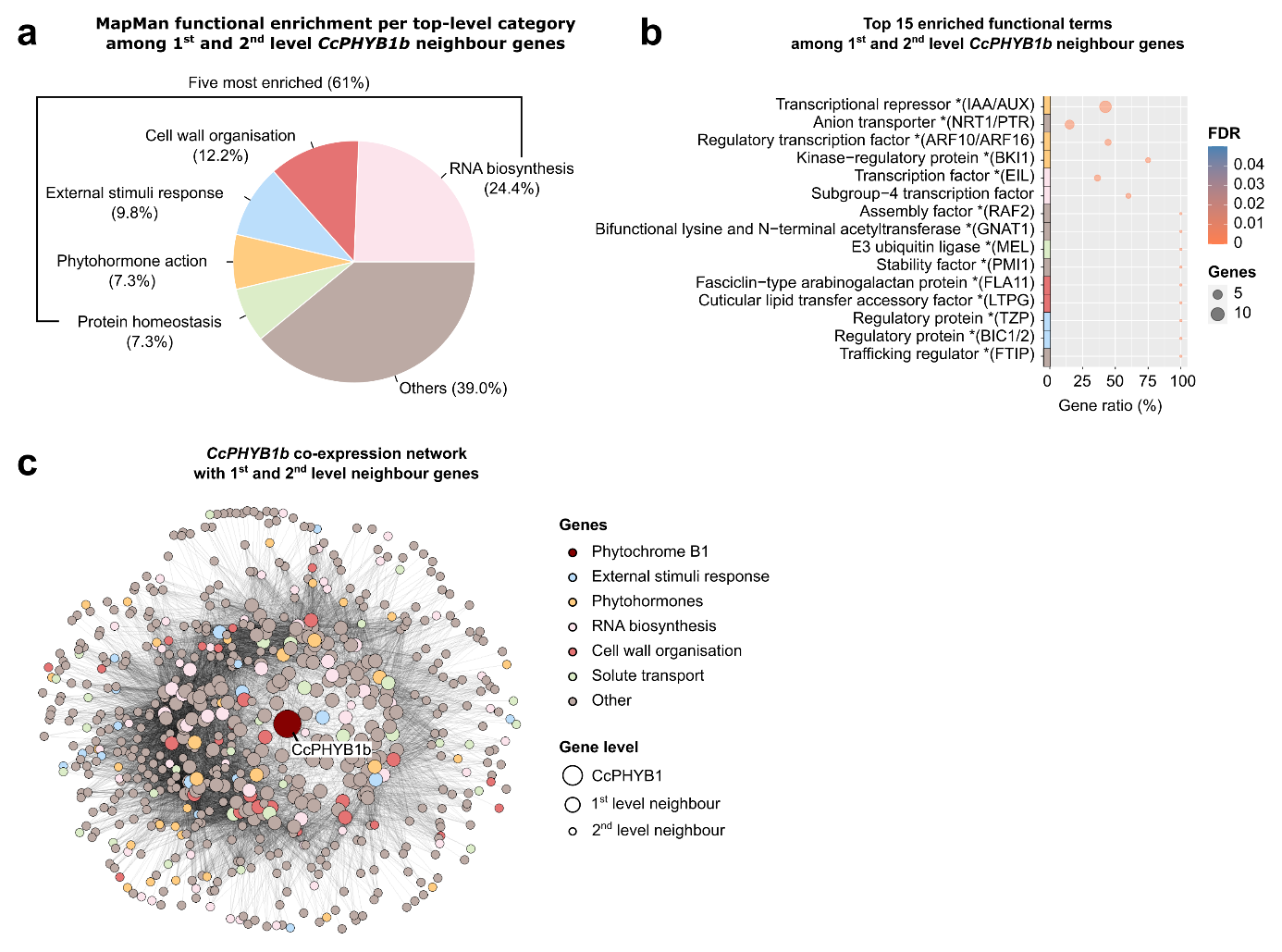


**Figure S5. Phytochrome B1b co-expression network.** (a) Schematic representation of enriched MapMan4 v.7.0 functional terms per top-level category within the *CcPHYB1b* co-expression network (1^st^ and 2^nd^ level neighbour genes). (b) Top enriched functional terms. Gene ratio refers to the number of genes with a term relative to the number of genes in the transcriptome with that term. (c) *CcPHYB1b* co-expression network, showing the connection of genes involved in (among others) phytohormone signalling, RNA biosynthesis, and cell wall organisation. Only shortest paths to phytochrome are displayed. The size of the nodes (genes) is proportional to their neighbourhood level.


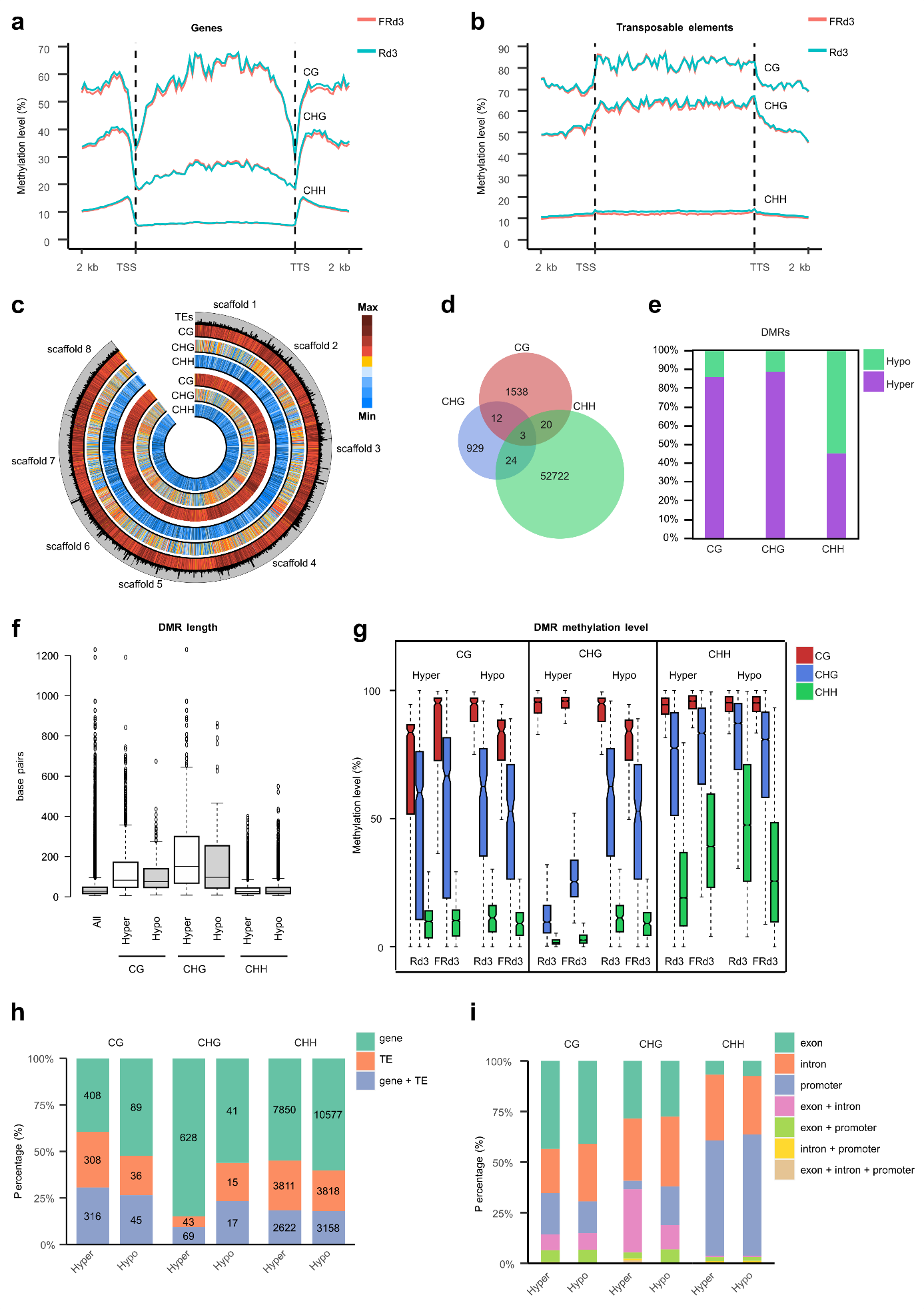


**Figure S6. Global DNA methylation.** (a) DNA methylation profile under red and far-red light in protein-coding genes from transcription starting site (TSS) to termination site (TTS). The length of each gene and its 2 kb flaking regions was normalized and divided into equal numbers of bins. Values are mean cytosine methylation levels per bin. (b) DNA methylation profile under red and far-red light in transposons. (c) Circular graph of DNA methylation levels in the first 8 scaffolds of the *C. campestris* genome in response to far-red (three outermost coloured tracks) or red light (three innermost coloured tracks) in CG, CHG, and CHH contexts. Red and blue colours in the tracks respectively represent highly and lowly methylated loci. The grey outer ring represents the abundance of transposons (TEs). (d) Venn representation of the number of DMRs and their overlap (≥1bp) in different contexts. (e) Proportion (%) of hyper-methylated (that is, with higher methylation level under far-red than red light) and hypo-methylated DMRs per context. (f) Average DMR length (bp) per context and methylation status. (g) Average cytosine methylation level (%) at hyper- and hypo-methylated DMRs (from left to right panel: CG, CHG and CHH DMRs) for each methylation context under red and far-red lights. (h) Categorisation per methylation context of the proportion (%) hyper- and hypo-methylated DMRs that are associated with protein-coding genes, transposons, or both. (i) Categorisation per methylation context of the proportion (%) hyper- and hypo-methylated DMRs that are associated with genomic features in protein coding genes.


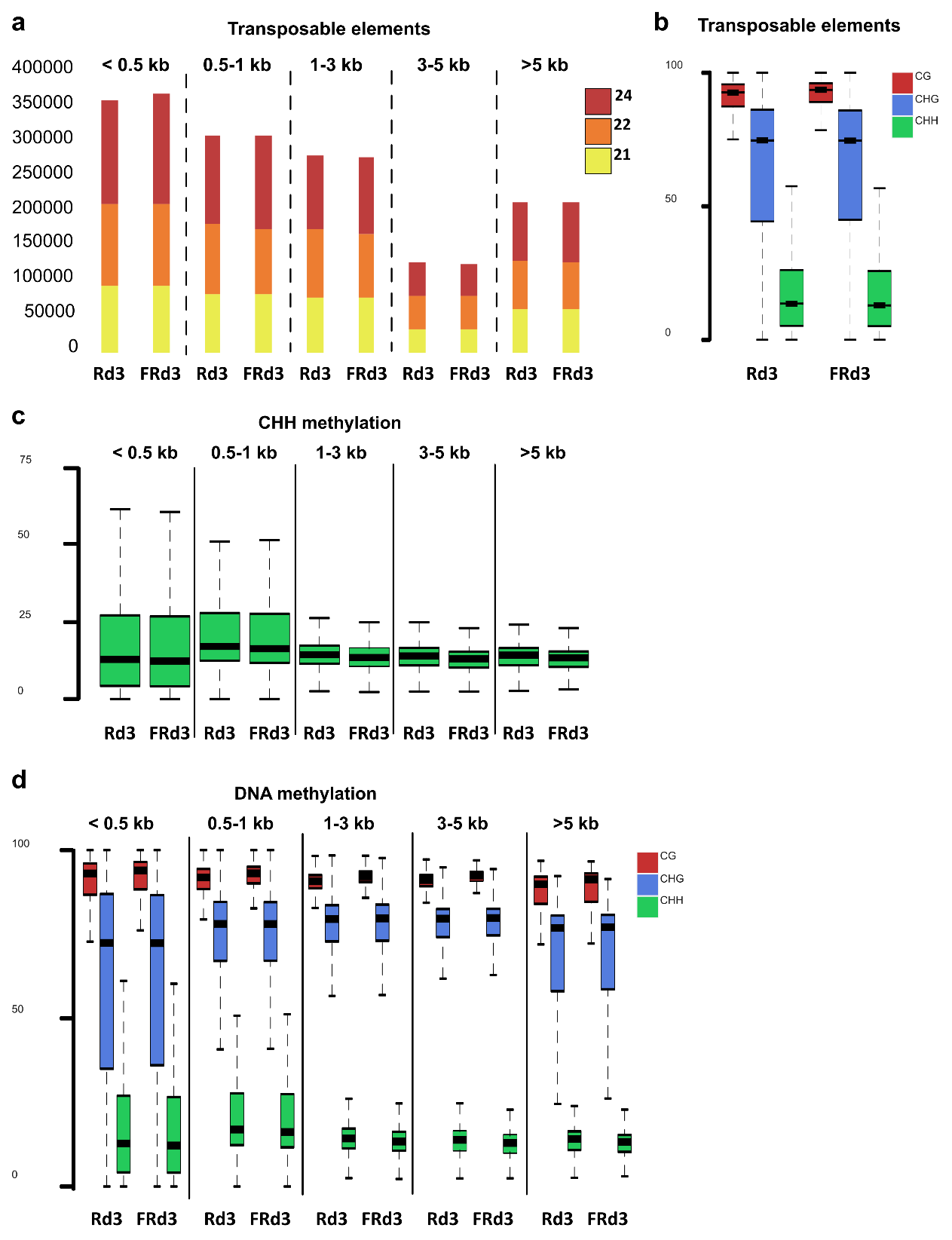


**Figure S7. Correlation between transposon size, accumulation of sRNAs and DNA methylation values.** (a) Accumulation of 21, 22 and 24-nt sRNAs as a function of transposon size and light condition. (b) Methylation level in transposons per context and light condition. (c) Methylation level in transposons in CHH context as a function of their size and light condition. (d) Methylation level in transposons per context as a function of their size and light condition.

**Table S1.** RNA mapping statistics.

Ctrl = control, R = red light, FR = far-red light, d1 = day 1, d3 = day 3.

| **Sample** | **Total reads** | **Uniquely mapped reads** | **%** | **Mulit-mapped reads** | **%** | **Unmapped reads** | **%** |
| --- | --- | --- | --- | --- | --- | --- | --- |
| FRd1_RNA_1 | 63475796 | 57178134 | 90.08 | 3953604 | 6.23 | 2344058 | 3.69 |
| FRd1_RNA_2 | 63460400 | 57259486 | 90.23 | 3744472 | 5.90 | 2456442 | 3.87 |
| FRd1_RNA_3 | 63235810 | 56851716 | 89.90 | 3971068 | 6.28 | 2413026 | 3.82 |
| FRd1_RNA_4 | 64932538 | 58516140 | 90.12 | 4022510 | 6.19 | 2393888 | 3.69 |
| FRd3_RNA_1 | 63916708 | 57553456 | 90.04 | 3561796 | 5.57 | 2801456 | 4.38 |
| FRd3_RNA_2 | 60696178 | 54814764 | 90.31 | 3305910 | 5.45 | 2575504 | 4.24 |
| FRd3_RNA_3 | 65340224 | 59007574 | 90.31 | 3531862 | 5.41 | 2800788 | 4.29 |
| FRd3_RNA_4 | 59959016 | 54170012 | 90.35 | 3313522 | 5.53 | 2475482 | 4.13 |
| Ctrl_RNA_1 | 65884260 | 59491114 | 90.30 | 3545566 | 5.38 | 2847580 | 4.32 |
| Ctrl_RNA_2 | 66499178 | 60020310 | 90.26 | 3472504 | 5.22 | 3006364 | 4.52 |
| Ctrl_RNA_3 | 60933468 | 55080444 | 90.39 | 3330322 | 5.47 | 2522702 | 4.14 |
| Ctrl_RNA_4 | 59920300 | 54231796 | 90.51 | 3261938 | 5.44 | 2426566 | 4.05 |
| Rd1_RNA_1 | 63968246 | 57544058 | 89.96 | 3867980 | 6.05 | 2556208 | 4.00 |
| Rd1_RNA_2 | 61177126 | 55269454 | 90.34 | 3327622 | 5.44 | 2580050 | 4.22 |
| Rd1_RNA_3 | 64032262 | 58035676 | 90.64 | 3475426 | 5.43 | 2521160 | 3.94 |
| Rd1_RNA_4 | 63244366 | 57089352 | 90.27 | 3743960 | 5.92 | 2411054 | 3.81 |
| Rd3_RNA_1 | 61465042 | 55151596 | 89.73 | 3438426 | 5.59 | 2875020 | 4.68 |
| Rd3_RNA_2 | 64639048 | 58358790 | 90.28 | 3360152 | 5.20 | 2920106 | 4.52 |
| Rd3_RNA_3 | 63354864 | 57130420 | 90.18 | 3244100 | 5.12 | 2980344 | 4.70 |
| Rd3_RNA_4 | 62597856 | 56390112 | 90.08 | 3335602 | 5.33 | 2872142 | 4.59 |

**Table S2.** Primer sequence pairs for RT-qPCR of the selected *C. campestris* phytochrome and reference genes with their amplicon sizes.

| **Accession** | **Gene** | **Forward (5'-3')** | **Reverse (5'-3')** | **Amplicon (pb)** |
| --- | --- | --- | --- | --- |
| Cc019681 | CcPHYAb | TTGCTTAGGGATGCTGCTTT | CAAGAGAAACTGCCCCTGAG | 235 |
| Cc031752 | CcPHYB1b | CGAGGCAAGCAATTCTAAGG | TCAACAGACAACCCAACCAA | 201 |
| Cc020059 | CcPHYB2a | ACAATCCTAAGGCCGTTGTG | ACTGACAACCCGACCAACTC | 169 |
| Cc003338 | SKIP/MAC6 | CGACAAGGGTCTCTTCAC | TAGCACCAGTCCTCTCAG | 173 |
| Cc001025 | RPT3 | TCATGGAGATGATCGACC | CAGAGAGATGCTAGAGTC | 185 |

**Table S3.** Small RNA mapping statistics.

R = red light, FR = far-red light, d3 = day 3.

| **Sample** | **Total sRNAs (18-28 nt)** | **Genome-mapped sRNAs**  **(18-28 nt, 2 mismatches)** | **Mappability (%)** |
| --- | --- | --- | --- |
| FRd3_RNA_1 | 11604991 | 11390533 | 98.15 |
| FRd3_RNA_2 | 9949356 | 9761782 | 98.11 |
| FRd3_RNA_3 | 8421342 | 8296900 | 98.52 |
| FRd3_RNA_4 | 11869586 | 11628009 | 97.96 |
| Rd3_RNA_1 | 12623508 | 12232697 | 96.90 |
| Rd3_RNA_2 | 10338986 | 10106581 | 97.75 |
| Rd3_RNA_3 | 9950678 | 9746033 | 97.94 |
| Rd3_RNA_4 | 11034556 | 10840207 | 98.24 |

**Table S4.** Differentially expressed miRNA families.

| **miRNA Family** | **Conservation** | **miRNA**  **direction** | **RPM**  **average Rs** | **RPM**  **average FRs** | **Ratio FRs_vs_Rs** | **t.test** |
| --- | --- | --- | --- | --- | --- | --- |
| miR166 | Conserved | Down | 34736.24 | 22464.56 | 0.65 | 0.00781 |
| miR319 | Conserved | Down | 23950.05 | 11471.34 | 0.48 | 0.04564 |
| miR165 | Conserved | Down | 96.41 | 27.10 | 0.28 | 0.00101 |
| miR164 | Conserved | Down | 85.81 | 13.80 | 0.16 | 0.03961 |
| miR390 | Conserved | Down | 70.94 | 5.72 | 0.08 | 0.01014 |
| miR894 | Conserved | Up | 6.09 | 11.71 | 1.92 | 0.03199 |
| miR170 | Conserved | Up | 53.47 | 101.73 | 1.90 | 0.01337 |
| miR171 | Conserved | Up | 312.78 | 517.68 | 1.66 | 0.03595 |
| miR168 | Conserved | Up | 93.66 | 142.50 | 1.52 | 0.00926 |
| miRNA28 | Species-specific | Down | 0.56 | 0.22 | 0.39 | 0.03606 |
| miRNA1 | Species-specific | Down | 24.53 | 4.45 | 0.18 | 0.00392 |
| miRNA13 | Species-specific | Up | 0.16 | 0.51 | 3.09 | 0.04179 |
| miRNA14 | Species-specific | Up | 8.92 | 13.40 | 1.50 | 0.01741 |

**Table S5.** DNA mapping statistics from BISMARK.

R = red light, FR = far-red light, d3 = day 3.

|  | **FRd3_DNA_1** | **FRd3_DNA_2** | **FRd3_DNA_3** | **Rd3_DNA_1** | **Rd3_DNA_2** | **RD3_DNA_3** |
| --- | --- | --- | --- | --- | --- | --- |
| Sequence pairs analysed in total: | 53897503 | 68279887 | 63900946 | 77344333 | 65708882 | 60287874 |
| Number of paired-end alignments with a unique best hit: | 42505054 | 53629444 | 49888632 | 60722251 | 51408293 | 47381586 |
| Mapping efficiency: | 78.9% | 78.5% | 78.1% | 78.5% | 78.2% | 78.6% |
| Sequence pairs with no alignments under any condition: | 6041581 | 8083342 | 7556468 | 9449290 | 7807766 | 7054141 |
| Sequence pairs did not map uniquely: | 5350868 | 6567101 | 6455846 | 7172792 | 6492823 | 5852147 |
| Sequence pairs which were discarded because genomic sequence could not be extracted: | 2683 | 3444 | 3070 | 3864 | 3101 | 3038 |
| CT/GA/CT: | 21249690 | 26826380 | 24950546 | 30357624 | 25709503 | 23689524 |
| GA/CT/CT: | 0 | 0 | 0 | 0 | 0 | 0 |
| GA/CT/GA: | 0 | 0 | 0 | 0 | 0 | 0 |
| CT/GA/GA: | 21252681 | 26799620 | 24935016 | 30360763 | 25695689 | 23689024 |
| Number of alignments to (merely theoretical) complementary strands being rejected in total: | 0 | 0 | 0 | 0 | 0 | 0 |
